# Predicting gene distribution in ammonia-oxidizing archaea using phylogenetic signals

**DOI:** 10.1093/ismeco/ycaf087

**Published:** 2025-05-23

**Authors:** Miguel A Redondo, Christopher M Jones, Pierre Legendre, Guillaume Guénard, Sara Hallin

**Affiliations:** Department of Forest Mycology and Plant Pathology, Swedish University of Agricultural Sciences, Box 7026, 750 07 Uppsala, Sweden; Département de sciences biologiques, Université de Montréal, C.P. 6128, succursale Centre-ville, Montréal, Québec H3C 3J7, Canada; National Bioinformatics Infrastructure Sweden, Science for Life Laboratory, Husargatan 3, 752 37 Uppsala, Sweden; Department of Cell and Molecular Biology, Uppsala University, Husargatan3, 752 37 Uppsala, Sweden; Department of Forest Mycology and Plant Pathology, Swedish University of Agricultural Sciences, Box 7026, 750 07 Uppsala, Sweden; Département de sciences biologiques, Université de Montréal, C.P. 6128, succursale Centre-ville, Montréal, Québec H3C 3J7, Canada; Département de sciences biologiques, Université de Montréal, C.P. 6128, succursale Centre-ville, Montréal, Québec H3C 3J7, Canada; Department of Forest Mycology and Plant Pathology, Swedish University of Agricultural Sciences, Box 7026, 750 07 Uppsala, Sweden

**Keywords:** phylogenetic modeling, trait imputation, elastic net regularization, phylogenetic eigenvectors, ancestral state reconstruction

## Abstract

Phylogenetic conservatism of microbial traits has paved the way for phylogeny-based predictions, allowing us to move from descriptive to predictive functional microbial ecology. Here, we applied phylogenetic eigenvector mapping to predict the presence of genes indicating potential functions of ammonia-oxidizing archaea (AOA), which are important players in nitrogen cycling. Using 160 nearly complete AOA genomes and metagenome assembled genomes from public databases, we predicted the distribution of 18 ecologically relevant genes across an updated *amoA* gene phylogeny, including a novel variant of an ammonia transporter found in this study. All selected genes displayed a significant phylogenetic signal and gene presence was predicted with an average of >88% accuracy, >85% sensitivity, and >80% specificity. The phylogenetic eigenvector approach performed equally well as ancestral state reconstruction of gene presence. We implemented the predictive models on an *amoA* sequencing dataset of AOA soil communities and showed key ecological predictions, e.g. that AOA communities in nitrogen-rich soils were predicted to have capacity for ureolytic metabolism while those adapted to low-pH soils were predicted to have the high-affinity ammonia transporter (*amt2*). Predicting gene presence can shed light on the potential functions that microorganisms perform in the environment, further contributing to a better mechanistic understanding of their community assembly.

## Introduction

Genomic information is essential for indirect trait-based approaches in microbial ecology, which can provide mechanistic explanations to ecological dynamics and ecosystem functions [1]. Microbial enzyme-encoding genes are often associated with the organismal functional traits and tend to be phylogenetically conserved [2, 3], which provides a foundation for phylogeny-based trait prediction [4–6]. We can therefore use massive amounts of environmental sequencing data to predict the probability of presence of certain microbial genes, thus inferring the potential functions that microorganisms perform in the environment. In this way, we can functionally characterize taxa whose genomes are not yet available or that represent a minor fraction in metagenomes. Phylogeny-based imputations of traits has generally used either ancestral state reconstruction by means of phylogenetic generalized least squares [6–8] or phylogenetic eigenvector maps [9–11]. In contrast to ancestral state reconstruction, phylogenetic eigenvectors offer the additional advantage of accommodating different modes of evolution, as well as the possibility of using phylogenetic signals together with abiotic factors when predicting traits or species distributions [12]. While phylogenetic eigenvectors have been used in a wide range of studies for macroorganisms, they have yet to be applied to microbial communities.

Among microorganisms, ammonia-oxidizing archaea (AOA) are an optimal group to evaluate the power of phylogenetic eigenvectors for predicting gene distribution. First, they are key players in the nitrogen cycle and inhabit most ecosystems on earth [13, 14]. Given their ecological relevance, AOA genomes and metagenome-assembled genomes (MAGs) are increasingly available, thus expanding our knowledge of the potential functions of archaeal genes [15–17]. Second, there is a coherence between the organismal phylogeny and that of the *amoA* gene encoding the ammonia monooxygenase subunit A, which has long been used as a marker gene for AOA in environmental studies [13, 18–21]. This has resulted in the availability of a global *amoA* phylogeny [21] that reflects the distribution of the organism across different earth environments. This adds to previous studies pointing at the niche specialization of certain AOA clades across varying levels of pH and other environmental properties [22–24]. Whether or not gene content can be predicted using the *amoA* phylogeny, thus providing a basis for a more mechanistic understanding of AOA community assembly, remains uncertain.

The aim of this work was to predict gene presence/absence in AOA using the *amoA* phylogenetic signal. To reach this goal, we updated a recent *amoA* gene reference phylogeny of AOA [21] by adding 160 highly complete AOA genomes and MAGs available in public databases. The updated phylogeny was then used together with phylogenetic eigenvector mapping [11] to predict the presence of a set of genes (Table 1) belonging to four functional categories selected from a comparative genomics study [16]. We validated the predictions using hold-out validations and compared them to estimations based on ancestral state reconstruction. Finally, we implemented the predictive approach on soil AOA communities obtained from a field study [25] and linked the predicted AOA gene presence to soil properties by means of simultaneous analysis of environmental characteristics, species distributions, and species traits [26]. This study demonstrates that phylogenetic eigenvector maps are useful for highly accurate predictions of gene distributions in AOA and can inform about their potential functions in the environment and the mechanisms underpinning community assembly.

**Table 1:** Genes selected for phylogenetic modeling, statistics for phylogenetic signal test, and validation results using phylogenetic eigenvectors and ancestral state reconstruction.

Gene	arCOG ID	Functional category (Kerou *et al*., [16])	Functional annotation/product (Kerou *et al*. [16])	Phylogenetic signal			Validation of gene predictions					
				*D* ^a^	*P* (*D* ≠ 1)^b^	*P* **(***D* ≠ 0)^b^	Phylogenetic eigenvectors			Ancestral state reconstruction		
							Accuracy (%)	Sensitivity (%)	Specificity (%)	Accuracy (%)	Sensitivity (%)	Specificity (%)
*amt2*	arCOG04397	N-metabolism	Ammonia permease	0.17	**<.001**	.174	93.3	99.1	55.8	91.3	96.2	57.7
*amt1*	arCOG04397	N-metabolism	Ammonia permease	0.06	**<.001**	.328	88.4	92.7	84.4	89.6	93.6	86.0
*amt-NC* ^c^	arCOG04397	N-metabolism	Ammonia permease	−0.38	**<.001**	.899	98.0	81.2	98.9	97.6	69.6	99.4
*ureC*	arCOG00698	N-metabolism	Urea amidohydrolase (urease), subunit alpha	0.36	**<.001**	**.003**	76.8	76.6	78.7	77.8	81.9	73.0
*metE*	arCOG01876	C/AA metabolism	Methionine synthase II (cobalamin-independent)	−0.36	**<.001**	.986	96.3	88.0	98.3	97.6	88.0	99.9
*metE2*	arCOG01877	C/AA metabolism	Methionine synthase II (cobalamin-independent)	−0.29	**<.001**	.957	95.0	85.3	97.4	96.5	84.7	99.3
*proDH*	arCOG06322	C/AA metabolism	Putative proline dehydrogenase	−0.08	**<.001**	.713	88.7	89.1	88.6	91.3	90.2	92.7
*rocA*	arCOG01252	C/AA metabolism	1-Pyrroline-5-carboxylate dehydrogenase	−0.14	**<.001**	.814	94.2	97.3	82.4	94.0	97.6	80.6
*cheA*	arCOG04403	Chemotaxis/motility	Chemotactic sensor histidine kinase CheA	0.11	**<.001**	.225	84.5	76.1	89.2	88.4	78.2	93.3
*cheY*	arCOG02391	Chemotaxis/motility	Chemotaxis response regulator CheY	0.05	**<.001**	.364	88.5	94.3	70.8	88.9	95.4	69.4
*cheY2*	arCOG02589	Chemotaxis/motility	Chemotaxis response regulator CheY	0.29	**<.001**	**.009**	79.1	88.3	66.3	82.7	91.7	70.3
*tadC*	arCOG01808	Chemotaxis/motility	Pilus assembly protein TadC	0.14	**<.001**	.131	82.1	84.8	80.5	82.3	84.9	80.4
*flaK*	arCOG02298	Chemotaxis/motility	Putative archaeal preflagellin peptidase FlaK	0.09	**<.001**	.218	85.5	87.1	84.8	84.6	88.8	81.5
*flaI*	arCOG01817	Chemotaxis/motility	ATPase involved in archaellum/pili biosynthesis	0.09	**<.001**	.247	86.1	91.5	81.2	85.3	89.8	81.0
*ipct*	arCOG00673	Environmental adaptation (osmotic regulation)	Bifunctional CTP:inositol-1-phosphate cytidylyltransferase/di-myo-inositol-1,3-phosphate-1-phosphate synthase	0.62	**.004**	**.001**	79.7	31.5	87.0	85.1	34.5	92.6
*nhaP*	arCOG01962	Environmental adaptation (osmotic regulation)	NhaP-type Na^+^/H^+^ and K^+^/H^+^ antiporter with a unique C-terminal domain/Cell volume regulation protein A	0.15	**<.001**	.197	89.8	95.9	50.5	90.5	96.4	52.8
*trk*	arCOG04145	Environmental adaptation (osmotic regulation)	Trk-type K^+^ transport system, membrane component	−0.21	**<.001**	.938	93.7	93.9	93.1	95.3	96.1	94.0
*cspC*	arCOG02983	Environmental adaptation (thermoadaptation)	Cold shock protein, CspA family	−0.21	**<.001**	.957	92.0	96.5	88.6	92.8	95.2	90.6
All genes (mean)							88.4	86.1	82.0	89.5	86.3	83.0

a *D* is the phylogenetic signal strength parameter, where values <0 indicates phylogenetic conservatism and values close to 1 indicate random distribution of gene value across the phylogeny.

b *D* ≠ 1 means departure from a random distribution, whereas *D* ≠ 0 means departure from a Brownian motion.

c *amt-NC* refers to a type of ammonia transporter gene that was found in this study to be present only in the *Nitrosocaldales* lineage (*NC*). Bold indicates *P*-value < 0.05.

## Materials and methods

### Update of the archaeal *amoA* reference phylogeny

To update the *amoA* phylogeny developed by Alves *et al*. [21], we first downloaded all genomes from isolates and MAGs from the National Center for Biotechnology Information (NCBI; https://www.ncbi.nlm.nih.gov/) and the Joint Genome Institute (JGI; https://genome.jgi.doe.gov/portal/), up to October 2021 using the search terms “Thaumarchaeota” and “Crenarchaeota,” since the search was done prior to the current taxonomy of the phylum harboring AOA (Nitrososphaerota). Sequences of 1527 archaeal genomes and MAGs were initially screened for the presence of *amoA* genes using HMMER (http://hmmer.org) and the translated alignment from Alves *et al*. [21] as seed alignment. Significant hits (*e*-value <10^−6^) were then aligned by amino acid to the original seed alignment using HMMER. An initial phylogeny of the significant hits, together with the 1190 sequences from Alves *et al*. [21] was generated from the nucleotide alignment using FastTree 2.1 [27] to ensure the correct identification of AOA *amoA* genes. It is important to note that we used a final dataset of 1190 sequences that Alves *et al*. [21] obtained as a result of excluding rogue sequences from their initial alignment of 1206 sequences. The alignment and tree were then inspected using the ARB software [28] to correct alignment errors and remove fragmented or poor quality (i.e. multiple “*N*”s) sequences, resulting in a total of 457 genomes and MAGs with *amoA* gene (Supplementary Table S1). We further discarded genomes/MAGs with <80% completeness or >5% contamination as determined by BUSCO [29] (archaea_odb10 database), as well as those with identical *amoA* sequences and the same presence/absence values of the selected genes (see below) to remove duplicate taxa. We further tested whether the *amoA* sequences were chimeras by using the reference-based chimera detection algorithm implemented in VSEARCH (v2.3.4) [30] software using the dataset of Alves *et al*. [21] as a reference and deleted 8 genomes/MAGs having chimeric *amoA* sequences. At the end, *amoA* sequences from 160 high-quality genomes and MAGs were added to the original alignment of 1190 sequences in Alves *et al*. [21], and the total alignment was used to build maximum likelihood phylogenies using the IQTree software [31], version 2.1.3. The tree search was carried out using three rounds of 20 independent tree searches, in which perturbation strength settings (*--pers* parameter in IQTree) of 0.1, 0.5, and 1.0 were used for each round, respectively. The resulting trees were checked individually, and the tree with both the highest likelihood and coherence with the Alves *et al*. [21] topology was selected as the final reference tree. Automatic model selection [32] resulted in GTR + F + R9 being selected as the best substitution model, and node support was determined by ultrafast bootstrap approximation and SH-aLRT tests using 1000 replicates for each support metric [33]. During the tree search one sequence from Alves *et al*., “NC-Alpha-OTU2” was the same as two other *amoA* sequences in our high-quality genomes and was thus removed by IQ-tree. Therefore, the final phylogenetic tree contained 1189 *amoA* sequences from Alves *et al*. [21] and the 160 *amoA* sequences from the high-quality genomes and MAGs we added. The display of the phylogenetic trees was produced using iTOL [34] and the “ggtree” package in R [35].

### Selection of genes for predictions and screening of genomes

We selected 18 genes for phylogenetic modeling (Table 1) based on the comparative genomic study by Kerou *et al*. [16]. These genes were (i) distributed across different AOA lineages to avoid genes only present in an isolated clade within the phylogeny and (ii) involved in pathways related to different ecological functions and especially relevant to soil AOA. The selected genes belonged to four categories: nitrogen metabolism (N-metabolism in figures) (*amt* and *ureC* genes), carbon and amino acid metabolism (C/AA-metabolism in figures) (*metE*, *proDH*, *rocA* genes), chemotaxis and motility (*cheA*, *cheY*, *flaK*, *flaI*, *tadC* genes), and environmental adaptation (*ipct, nhaP*, *trk,* and *cspC* genes). We downloaded the orthologous gene protein alignment from each of these genes from the EggNOG 5.0 database (http://eggnog5.embl.de) using the arCOG ID reported by Kerou *et al*. [16]. When more than one arCOG was provided, we used each of them separately. With the alignments obtained from the EggNOG database, the 160 AOA genomes/MAGs were screened for the presence/absence of each gene using HMMER (*e*-value <0.01). To ensure that gene hits were accurate, we retrieved the protein sequences found on the genomes/MAGs, aligned them with the reference protein alignment from the EggNOG database, and constructed phylogenies using the fastTree2 software [27], version 2.1.11. The location of the hits relative to the reference sequences within the phylogenies was examined to make sure that they were in fact hits of the searched genes and not artifacts or other homologs with different functions.

In the specific case of the ammonium transporter gene (*amt*), and due to its ecological relevance, the gene phylogeny was used to further classify the hits as Amt2 (high-affinity) or Amt1 (low-affinity) type of transporter [36–38]. The hits falling together with *Nitrosocosmicus* taxa in the phylogeny were assigned the low-affinity transporter type (Amt1), as all sequenced *Nitrosocosmicu*s taxa encode uniquely one low-affinity Amt [39–42]. It should be noted here that other studies have used the reverse nomenclature, in which Amt1 and Amt2 are defined as high- and low-affinity transporters, respectively [43–45]. To clarify these inconsistencies in the nomenclature, we screened the Amt hits of our study for the primers used by Nakagawa and Stahl [37] for both types of transporters and found that the *amt2* primers from Nakagawa and Stahl [37] matched the genes that were defined as *amt1* in Offre *et al*. [43] and vice-versa. Therefore, we refer here to the gene encoding the high-affinity transporter as *amt2* [36, 37], which corresponds to the *amt1* in Offre *et al*. [43]. The procedure of checking the *amt* hits using phylogenies allowed us to find a third type of ammonium transporter uniquely present in the *Nitrosocaldales* (*NC*) lineage (Supplementary Text S1; Supplementary Figs S1 and S2; and Supplementary Table S2). The gene names and arCOG references are provided in Table 1.

### Test of phylogenetic signal for selected genes

We tested the phylogenetic signal of each gene using the phylo.d function from the caper package [46], running 1000 permutations. This method determines the strength and the statistical significance of the phylogenetic signal for binary traits, i.e. in this study binary trait refers to the presence/absence of a specific gene, compared to random and Brownian motion distributions of the trait [47]. The resultant parameter (*D*) equals 1 when the binary trait has a random distribution across the phylogeny, and 0 if under Brownian motion. *D* values can be <0 if the trait distribution is more clustered than expected under Brownian motion, and >1 if it is more overdispersed than expected by random. The phylo.d function tests *D* for significant departure from 1 when the trait is phylogenetically conserved, and a significant departure from 0 when the trait does not follow a Brownian evolutionary model.

### Prediction of gene presence/absence using phylogenetic eigenvectors and comparison to ancestral state reconstruction

The input for the predictions of gene presence was the final *amoA* phylogenetic tree and the matrix of presence/absence of the 18 genes across the 160 AOA genomes/MAGs. We then used phylogenetic eigenvectors obtained from the phylogenetic tree and compared the prediction results to those obtained by ancestral state reconstruction. In both cases, we predicted the probability of the presence of each gene in the taxa across the *amoA* phylogeny that had unknown genomes.

The phylogenetic eigenvector-based predictions were done in three steps: (1) decompose the phylogenetic tree into eigenvectors, (2) fit and regularize individual predictive models for each gene using the eigenvectors as the descriptors, and (3) estimate the presence/absence of the genes from the models of Step 2 on the taxa with unknown genomes given their locations in the phylogeny. To decompose the phylogenetic tree into eigenvectors (Step 1), we used R package MPSEM [48]. The input for the MPSEM package is the phylogenetic tree containing all tips, i.e. taxa with and without gene information. The MPSEM package calculates an influence matrix using only the tips of the tree for which there is information about presence/absence of genes by pruning from the tree the taxa without gene information. From the influence matrix, phylogenetic eigenvectors were obtained by singular value decomposition. When calculating the phylogenetic eigenvectors, we fixed argument *a* = 0 to assume a Brownian motion evolution of all genes. In Step 2, the phylogenetic eigenvectors were then used as fixed factors in a multiple logistic regression whose coefficients were regularized using elastic net regularization. For model regularization, we used the R package glmnet [49] with arguments family = “binomial” and *α* = 0.5 to use same amounts of both *L*_1_ (LASSO) and *L*_2_ (ridge regression) shrinkage. The penalization hyper-parameter (*λ*) was tuned using leave-one-out cross validation within the training dataset and choosing the *λ* value that provided the highest accuracy of the predictions. The outcome of Step 2 is a regularized model that predicts the probability of gene presence given the eigenvectors’ values. We classified the probabilities of the predictive model into presence or absence by choosing a threshold that maximizes both true-positive (sensitivity) and true-negative rate (specificity) of the predictions. For this, we created ROC curves using the R package pROC [50] with the function roc and used the function coords to select a threshold for classification that would render the highest Youden’s *J* statistic, where *J* = sensitivity + specificity − 1. Probabilities that had values equal to or greater than the selected threshold were classified as presence, whereas probabilities lower than the threshold were classified as absence. The final output of Step 2 is a model with tuned *λ* and classification threshold parameter that predict the gene presence for a taxon given its phylogenetic eigenvector scores. We then obtained the eigenvector scores for taxa with unknown genomes and used them on the model of Step 2 to predict the gene presence. Function getGraphLocations from the MPSEM package places the taxa with unknown genomes in the initial influence matrix of Step 1 and function Locations2PEMscores obtains the phylogenetic eigenvector scores. It is important to note that the influence matrix and phylogenetic eigenvectors are not obtained using all tips of the tree, but only the ones with known gene information. For taxa with unknown information, we calculated the eigenvector score values, which are projections of new influence matrix coordinates on the eigenvectors obtained from the initial influence matrix (see Guénard *et al*. [11], for details on this procedure). Thus, the number of phylogenetic eigenvectors of the training dataset, and therefore the number of coefficients of the predictive model, is independent of the number of taxa to be predicted. We provide the code that can be used for analysis of other datasets by providing the phylogenetic tree and its associated presence/absence table with and without missing values in the input data.

The ancestral state reconstruction was done with R package picante R [51]. We used the phyEstimateDisc function to predict the genes of the AOA taxa with unknown genomes. In this procedure, for each taxon with unknown gene presence data, the phylogenetic tree is rerooted on the most recent ancestor common to the unobserved taxon and the rest of the phylogeny. The gene presence or absence of the unobserved taxon is then estimated from the ancestral state reconstruction of the root of the rerooted phylogeny [7, 52]. The function phyEstimateDisc provides a trait state, i.e. presence or absence, for a given threshold (default = 0.5), as well as a value for the statistical support of the state.

### Validation of the predictive models

We validated the predictions of each gene using a 20% hold-out validation. This procedure consists of randomly removing 20% of the initial dataset before creating the predictive model and validating it on the removed samples. We repeated this procedure 500 times, always randomizing the taxa included in the held-out dataset. For each gene, we obtained the mean accuracy, sensitivity, and specificity of the prediction. We also varied the proportion of taxa to be held out in the validations to 30% and 40% and obtained accuracies of 87.1% and 86.4%, respectively (compared to >88% accuracy at 20% hold out). We could not increase the proportion of data to be held out because many genes were class unbalanced. Both sensitivity and specificity were obtained using R package pROC.

We validated the accuracy of the predictions at the genome/MAG level using leave-one-out cross validation, in which we deleted each genome/MAG from the dataset in turn and used the rest of the genomes to predict the gene content of the previously removed genome or MAG.

### Implementation of predictive modeling on natural communities: a case study

To illustrate how predicting AOA gene distribution could link community composition, potential functions, and environmental properties, we used data from a previous study that characterized AOA communities by *amoA* amplicon sequencing on 50 sampling points across an agricultural area in which soil properties were measured (see Enwall *et al*. [53] and Jones and Hallin [25], for more information). The study site is a 44 ha farm divided into 14 fields, with sampling points taken at 51 locations throughout the fields based on environmental gradients identified in a previous study [54]. We deleted one of the locations (S17) because it lacked data on soil properties. We predicted the presence/absence of the 18 genes on the AOA communities and linked the gene composition with the soil properties that were reported by Enwall *et al*. [53].

To predict the gene presence of the AOA members of the soil communities, we used the representative sequences of the operational taxonomical units (OTUs) (162 in total) from the study of Jones and Hallin [25]. In that study, OTUs were obtained by clustering the postprocessed reads at 97% nucleotide similarity using the UPARSE algorithm [55]. We then placed the *amoA* sequences of each OTU on the reference phylogeny using the EPA-ng [56] (accessed at https://github.com/pierrebarbera/epa-ng) and gappa software [57] (accessed at https://github.com/lczech/gappa). The output of these analyses is a phylogenetic tree containing all sequences of our reference phylogeny and the representative OTU sequences to be predicted as grafted leaves. We used this phylogenetic tree and grafted leaves to implement the phylogenetic eigenvector modeling described above. The output was a matrix with presence/absence of all 18 genes for each OTU in the study.

We studied the link between the predicted genes (Q matrix) and the soil properties (R matrix) mediated by community composition (L matrix) by performing a RLQ analysis followed by a univariate fourth-corner analysis [26, 58, 59]. RLQ is an ordination method that displays the covariation of traits, i.e. the predicted gene presence, and environmental properties providing site and species (hereinafter OTUs) scores, and a global test for significance. The fourth-corner correlations, on the other hand, provide tests of single associations between predicted gene presence and environmental properties. The two methods are complementary and can be performed sequentially. For the RLQ analysis and following Dray *et al*. [26], we calculated three separate ordinations using the ade4 package for R [60]: (1) a correspondence analysis for the community data, i.e. OTU table, using the function dudi.coa; (2) a combination of principal component analysis and multiple correspondence analyses using the environmental data matrix, i.e. soil properties, after standardization using the function dudi.hillsmith; and (3) a principal component analysis for the predicted genes presence/absence without standardization using the function dudi.pca. The three ordinations were analyzed together using the rlq function of the ade4 package for R [60]. The RLQ analysis was tested for significance using the randtest function of the ade4 package with argument modeltype = 6 for the permutation test. This model performs two sequential permutational tests, a first one testing the link between OTUs distribution and environmental conditions (Model 2), and a second testing the link between OTUs distribution and predicted gene presence (Model 4). When both tests are significant, the highest of the two *P*-value provides the statistical significance for the global tests of association between gene distribution and environment [61].

To test which specific gene was associated with each soil property, we performed a fourth-corner analysis using the fourth-corner function from the ade4 package, with modeltype = 6, 99 999 permutations, and “false discovery rate” as the multiple testing correction method for the *P*-value.

## Results

### Congruence between *amoA* phylogeny and content of genes for predictions

**Figure 1:**
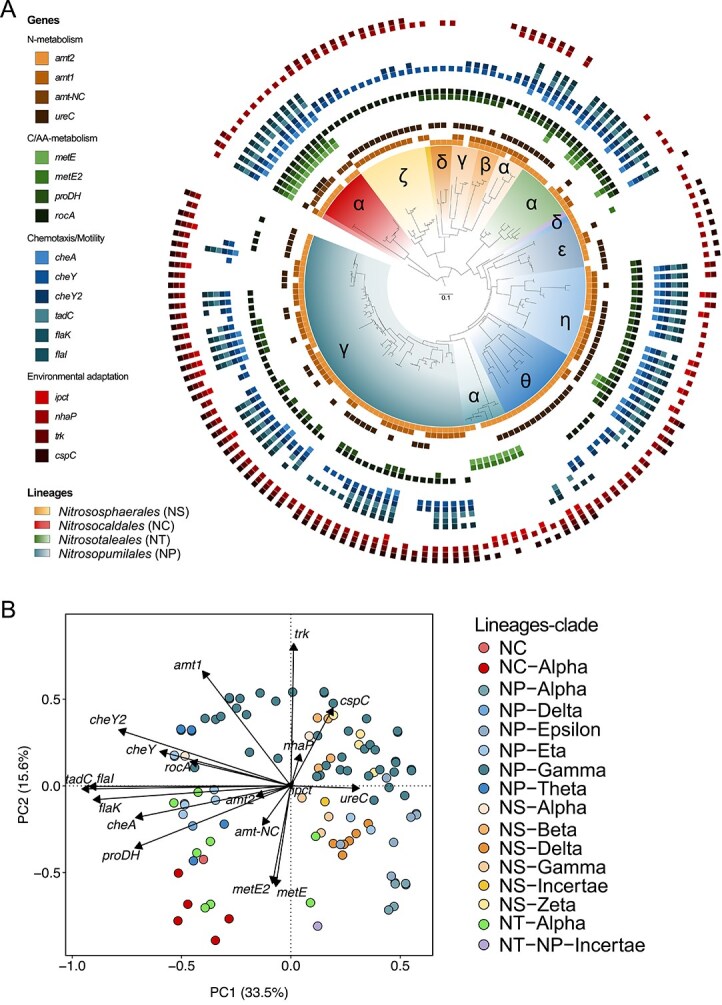
Distribution of the 18 selected genes in AOA genomes. (A) The presence/absence of 18 ecologically relevant genes (Table 1), across a phylogeny of 160 AOA isolate genomes and MAGs. Outer rings depict presence (filled squares) and absence (no squares) of specific genes. Clades within each AOA lineage are denoted by Greek letters. The phylogeny of 160 genomes is the result of pruning taxa that lack genomic information from the updated reference phylogeny. For bootstrap node support of the lineages of the *amoA* updated phylogeny, see Supplementary Fig. S3. (B) Principal component analysis of the 160 genomes and MAGs based on a presence–absence matrix for the 18 selected genes.

To perform the phylogenetic eigenvector-based predictions, we first updated the archaeal *amoA* gene reference phylogeny from Alves *et al*. [21], which now contains 1349 unique *amoA* sequences, including those from 160 highly complete AOA genomes of isolates and MAGs distributed across most *amoA* lineages (Supplementary Fig. S3). After screening the genomes and MAGs for the presence/absence of the genes selected for modeling (Table 1), we found that *Nitrososphaerales* (*NS*) taxa overall lacked genes associated with motility (*tadC*, with the exception of *NS*-α), osmotic regulation (*trk*), and thermoadaptation (*cpsC*)*.* The *metE* gene, responsible for methionine synthesis in energy-limiting environments [16], was found in *Nitrosopumilales* (*NP*) clades associated to deep sea waters (*NP*-α and -θ), as well as in *NC* and *Nitrosotaleales* (*NT*) (Fig. 1A). Genes encoding the high- and low-affinity ammonium transporters (Amt2 and Amt1, respectively [36, 37], see details in Methods) were identified based on their positions within a phylogenetic tree of translated Amt sequences [43]. When doing this, we identified a gene encoding a novel variant of the ammonia transporter protein specifically associated with the *NC amoA* lineage, hereafter referred to as Amt-NC. All taxa having the *amt-NC* gene also had the *amt2* (Fig. 1A). We found two subgroups of the ammonium transporter gene *amt-NC*, hereinafter *amt-NC.1* and *amt-NC.2* (Supplementary Fig. S1), which corresponded to the groups into which Luo *et al*. [17] divided the *NC* lineage based on the concatenation of 122 archaeal genes (Supplementary Text S1). The amino acid composition of the Amt-NC was identical to the Amt types described by Offre *et al*. [43] at the ammonium-binding sites, i.e. they contained the same histidine lining the transporter pore [43] and had the same amino acids in several conserved loci (Supplementary Fig. S2). However, it differed in several loci across the regions described by Offre *et al*. [43] (Supplementary Fig. S2). Our findings of a potentially novel variant of the ammonia transporter are solely based on phylogenetic alignment and differences in amino acid composition. Future studies should validate its function and examine if there are functional differences between the proteins encoded by the *amt-NC* and *amt1* and *amt2* genes.

All selected genes were phylogenetically conserved (Table 1). The distance between the 160 isolate genomes and MAGs in a principal component ordination including all 18 genes reflected the lineage classification of the AOA taxa (Fig. 1B), supporting a coherence between the *amoA* phylogeny and the overall gene content of available genomes and MAGs.

### Phylogeny-based predictions of selected genes

**Figure 2:**
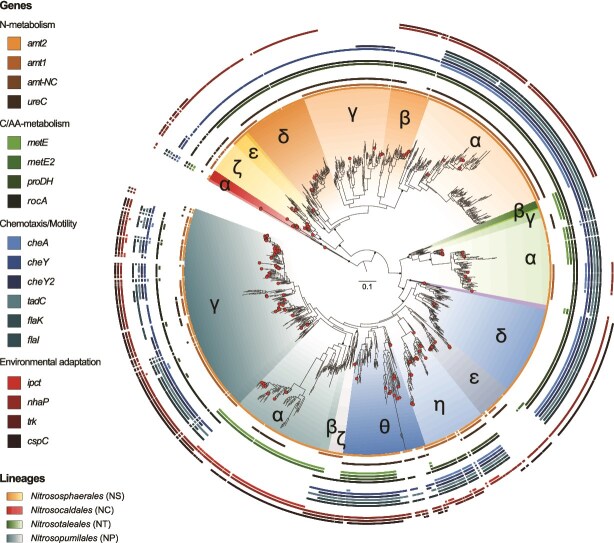
Predictions of gene presence/absence across the archaeal *amoA* reference phylogeny using phylogenetic eigenvectors. Outer rings depict predicted presence (filled squares) and absence (no squares) of specific genes (see Table 1 for definition). Circular markers at the tips represent the isolate genomes and MAGs with >80% completeness and <5% contamination that were used to train the predictive model. Clades within each AOA lineages are denoted by Greek letters. Nodes with >80 SH-aLRT and >70 ultrafast bootstrap are indicated by solid points, whereas those with >80 SH-aLRT support only are indicated by lighter-shaded points. For better visualization, we only display bootstrap values for lineages and clades. See Supplementary Fig. S3 for all bootstrap values across deeper nodes.

We used the gene content and phylogenetic relatedness of the 160 genomes and MAGs to build predictive models of gene presence. For each of the 18 genes, we used elastic net regularized regressions, with the phylogenetic eigenvectors as predictors and the presence of each gene as response. The models with optimized *λ* penalization and classification threshold parameters (Supplementary Table S3) were then used to predict gene presence across the *amoA* reference phylogeny using the phylogenetic eigenvector scores of unobserved taxa as input. The predicted presence of most genes varied across and within *amoA* lineages (Supplementary Table S4). For example, the gene encoding the high-affinity ammonia transporter (*amt2*) was predicted to be present in nearly all lineages except *NS*-ζ. In contrast, the low-affinity ammonia transporter (*amt1*) and urease (*ureC*) genes were predicted to be within most *NS* clades yet were more unevenly distributed or absent across *NT* and *NP* clades (Fig. 2; Supplementary Tables S4 and S5). Regarding genes involved in carbon and amino acid metabolism, the gene responsible for B-12 independent methionine synthesis (*metE*) was predicted to be present in all *NC* and in most *NT* taxa. Across *NP* clades, *metE* was predicted to be present in *NP*-α and -β, as well as in most *NP*-θ and some *NP*-η, -ε, and -γ (Fig. 2; Supplementary Tables S4 and S5). Genes involved in chemotaxis and motility showed the highest within-clade variation, where presence of genes related to archaellum formation (*flaK* and *flaI*) varied between taxa of the *NP*-γ clade (Fig. 2; Supplementary Tables S4 and S5), while nearly all lineages except *NP*-ε and -α were predicted to have the *cheY* gene, encoding a response regulator associated with chemotaxis. Regarding environmental adaptation, genes associated with osmotic regulation (*nhaP*, *trk*) and thermoadaptation (*cpsC*) were also predicted to be present more often in *NP* clades and less often in *NS* (Fig. 2; Supplementary Tables S4 and S5). The presence of *ipct* gene was mostly restricted to taxa of the *NP*-α clade. A similar pattern of gene presence was obtained using ancestral state reconstruction (Supplementary Fig. S4; Supplementary Tables S6 and S7); a co-inertia analyses, i.e. a test of collinearity between two matrices, performed on phylogenetic eigenvector- and ancestral state reconstruction-based predictions had a *R*^2^ of 0.73.

The phylogenetic eigenvector-based predictions of gene presence for all genes resulted in an average of 88.4% accuracy, 86.1% sensitivity, and 82% specificity based on a 20% hold-out-validation (Table 1). Ancestral state reconstruction of gene presence resulted in similar levels of accuracy, sensitivity, and specificity of predictions (89.5%, 86.3%, and 83%, respectively; Table 1). For both methods, the predictive accuracy increased linearly with the strength of the phylogenetic signal (*R*^2^ = 0.71 and 0.68 for phylogenetic eigenvectors and ancestral state reconstructions, respectively, Supplementary Fig. S5).

#### Link between predicted genes and soil properties: a case study

**Figure 3:**
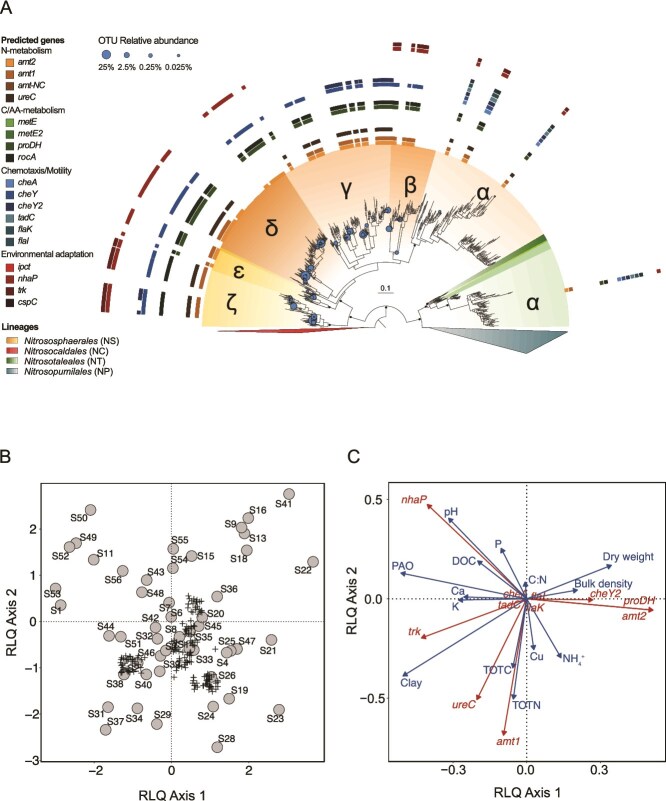
Association of predicted genes of AOA communities with soil properties across 44 ha of arable land. (A) Our updated *amoA* reference phylogeny with the placements of the 162 operational taxonomical units (OTUs) as grafted leaves. For visualization purposes, the relative abundances of the OTUs are displayed as circles on the nodes where they were placed. The circle size is proportional (fifth root) to their relative abundance. Outer rings depict predicted presence (filled squares) and absence (no squares) of specific genes for each OTU (see Table 1 for definition). None of the OTUs belonged to *NP* nor *NC*, and therefore both lineages are collapsed in the tree. Clades within each AOA lineage are denoted by Greek letters. Nodes with >80 SH-aLRT and >70 ultrafast bootstrap are indicated by solid points, whereas those with >80 SH-aLRT support only are indicated by lighter-shaded points. For better visualization, we only display bootstrap values for lineages and clades. See Supplementary Fig. S3 for all bootstrap values across deeper nodes. (B) Biplot of the RLQ analysis displaying scores of 50 sites (S1–S50) and 162 operational taxonomical units (OTUs) in circles and plus (+) signs, respectively. OTU symbols were jittered for visualization purposes. (C) Biplots of the RLQ analysis displaying association between predicted genes (red) and observed soil properties (blue). PAO refers to potential ammonia oxidation rates. The genes whose predicted values were all 0 or 1 were not included in the RLQ analysis. The global *P*-value associated with the RLQ analysis was 0.02.

To exemplify how predictions of gene presence/absence can contribute to indirect trait-based studies, we placed the *amoA* sequences of the members of 50 AOA communities from arable land in the updated *amoA* reference phylogeny, predicted the gene presence among these *amoA*-based OTUs (Fig. 3A), and tested the link between predicted genes and soil properties by performing RLQ [58] and fourth-corner [59] analyses. The first RLQ axis showed that OTUs predicted to have genes encoding the high-affinity ammonia transporter (*amt2*), chemotaxis response (*cheY*2), and proline dehydrogenase (*proDH*) genes yet lacking the *nhaP* gene were more associated with most of the sites with lower pH (pH = 5.7–6.0 in sites S19, 21, 23, and 28; Figs. 3B and C). The second RLQ axis highlighted two specific genes, i.e. the low-affinity ammonia transporter (*amt1*) and the *ureC*, which were most closely associated to sites with higher levels of total nitrogen and carbon, and more moderate soil pH (pH = 6.1–6.2 in sites S29, 31, 34, 37; Figs. 3B and C). When testing the univariate associations of each gene and soil property using the fourth-corner approach, significant correlations were only found before correcting the *P*-values for multiple testing (Supplementary Fig. S6).

## Discussion

Predicting gene presence can inform about key potential functions that microorganisms perform in the environment without having access to their full genomes. In this study, we predicted the presence of a set of genes of AOA with >88% accuracy using the *amoA* phylogenetic signals implemented in phylogenetic eigenvectors. The overall distribution of our isolate genomes and MAGs across the phylogeny supports the reliability of these predictions, particularly for AOA belonging to clades with good genome representation (see Supplementary Table S8). While genome and MAG incompleteness may limit inferences of potential microbial functions [62], we did not find any association between predictive accuracy and genome completeness (Supplementary Fig. S7A). We only observed a weak trend suggesting that the presence of genes in highly complete genomes was predicted with lower sensitivity and higher specificity than in less complete genomes (Supplementary Fig. S7B and C). Thus, including more incomplete genomes in the training dataset could increase false-negative predictions.

The high prediction accuracy can be explained by the strength of the phylogenetic signal of the selected genes. All the screened genes were conserved phylogenetically, in line with previous studies [16, 19], and the strength of the phylogenetic signal was positively correlated with the accuracy of the predictions [5, 6]. For example, the *amt-NC* gene had one of the strongest phylogenetic signals and displayed the highest prediction accuracy, while the *ureC* gene had one of the weakest signals and displayed the lowest accuracy. The differences in phylogenetic signal between genes are likely to be the evolutionary result of niche specialization. The *amt-NC* variant of the *amt* gene, described here for the first time, encodes an ammonia transporter that is present only in *Nitrosocaldus* AOA inhabiting thermal waters (Supplementary Text S1), and is therefore localized in a single clade within the *amoA* phylogeny. By contrast, the *ureC* gene tends to be phylogenetically dispersed. In agreement, soil AOA thrive in conditions in which ammonia is supplied slowly through mineralization of organic matter [63], and urease genes are abundant in Nitrososphaerota communities in oligotrophic marine environments [64]. The overall high accuracy of the predictions may also be the result of the availability of AOA genomes and MAGs across the *amoA* phylogeny. Accordingly, predictions on genomes and MAGs belonging to the *NS*-ζ and *NC*-α, *NP*-ε, and *NP*-α clades had the highest accuracies, and these AOA lineages were well-represented by genomes with close phylogenetic relatedness to the within-clade available relatives [5, 65] (Supplementary Table S8). Phylogenetic modeling of potential microbial traits should be therefore restricted to groups of organisms with good genomic representation in the databases [4, 66].

Predictions of gene distribution can provide a mechanistic understanding of community assembly in the environment. By implementing the gene presence predictions in multivariate models of AOA communities in soils with varying physical and chemical properties, we show that AOA communities adapted to low pH soils are more likely to have the high-affinity ammonia transporter (*amt2*), chemotaxis gene *cheY2*, and proline dehydrogenase gene *proDH*. This makes sense given the low availability of ammonia at low pH and is in line with previous studies showing down-regulation of bacterial chemotaxis genes at high pH and involvement of proline metabolism in abiotic stress response [67, 68]. By contrast, high pH sites were associated with OTUs predicted to have the *nhap* gene encoding a protein related to Na^+^/H^+^ antiporters, which are more important for homeostasis under neutral or alkaline conditions [69]. Resource rich sites with high amounts of total nitrogen and soil organic carbon, located in areas with higher influx of nitrogen via biological N_2_ fixation [22], were related to the increase of relative abundance of AOA taxa with predicted potential for ureolytic metabolism (*ureC*). Accordingly, nitrogen addition may increase the abundance of *ureC* genes [70]. Higher potential ammonia oxidation activity was linked to taxa with the low-affinity ammonium transporter. This could be because the high ammonium concentrations used in the activity assay favored the ammonia oxidizers with low ammonia affinity or those that prefer inorganic to organic N sources, including ammonia-oxidizing bacteria [71]. Overall, our predictions on soil AOA communities show that the combination of gene presence predictions with RLQ and fourth-corner analyses can shed light on mechanisms of community assembly. This approach can be expanded to microbial groups other than AOA to decipher ecological dynamics, given a well curated reference database and phylogeny to which either amplicon, metagenomic, or metatranscriptomic data can be mapped.

Phylogenetic eigenvector mapping can complement the broadly used ancestral state reconstruction when performing phylogeny-based modeling. We show that both methods have similar values of accuracy, sensitivity, and specificity of the predictions, and accuracy values between these two methods across all genes were highly correlated (*R*^2^ = 0.97). Based on co-inertia testing, both predictions across the phylogeny were associated with a high *RV* coefficient value, i.e. a correlation metric between two multivariate sets of variables [10], of 0.73. When setting the threshold for classifying probabilities at 0.5 in our phylogenetic eigenvector models, which is the default for ancestral state reconstruction in the R package picante [51], the *RV* increased to 0.78. The use of either phylogenetic eigenvector maps or ancestral state reconstruction depends on the role of phylogeny in the analyses [72, 73]. When predicting presence of gene using only phylogenetic signals, both methods give similar results, with ancestral state reconstruction not requiring the extra step of selecting or regularizing a model [6]. Regularizing a model may particularly be challenging when dealing with uneven class distribution. For example, when predicting the presence of the amt-NC gene across the phylogeny, the optimized *λ* hyper-parameter of the elastic net was 0.31, and all coefficient estimates shrank to 0, i.e. all taxa in the phylogeny would have the same probability of gene presence. Although modifying the *λ* value would render the same class predictions between phylogenetic eigenvectors and ancestral state reconstruction, the latter method does not rely on hyper-parameter tuning and therefore more useful on cases with very few presences or absences of specific genes. On the other hand, phylogenetic eigenvectors can be used when modeling phylogenetic signal together with other factors, e.g. abiotic variables or gene co-occurrences, which is useful when estimating traits on partially incomplete databases [74, 75]. Although in this study we use phylogenetic eigenvector maps to predict the probability of gene presence and not functions themselves, our approach could be used to predict high-level functional trait activities when information about them is present in the input data. In addition, the procedure used by the MPSEM R [48] package to calculate phylogenetic eigenvectors can use phylogenetic networks as input. Thus, phylogenetic eigenvectors can potentially be used for microorganisms for which horizontal gene transfer occurs, as well as for organisms that undergo reticulate speciation or hybridization [76]. Finally, the sets of latent descriptors produced by phylogenetic eigenvector mapping can be used with other modeling approaches, such as support vector machines, gradient boost machines, or artificial neural networks. Although some studies report a better performance of ancestral state reconstruction over phylogenetic eigenvectors [77], perhaps because of model selection issues, the present study shows that both can provide similar accuracies, while phylogenetic eigenvector maps constitute a more versatile tool for phylogenetic modeling.

To conclude, we show that we can move toward gene distribution predictions in microbial ecology. Whereas the absence of many microbial genomes can limit the implementation of trait-based studies in microbial ecology, many microbial genes are conserved phylogenetically, and their presence can be predicted using such tools as phylogenetic eigenvectors and ancestral state reconstruction. Predictive modeling of potential microbial functions can provide useful information to understand how evolution shapes the genetic content of microorganisms, how that determines their distribution in the environment, and how that ultimately may impact ecosystem functions.

## Supplementary Material

Redondo_et_al_supplementary_information_ismecom_revised3_ycaf087

Supplementary_table_1-5-7_ycaf087

Supplemental_fig3_ref_phylogenytree_ycaf087

AOA_amoA_redondo2024_ycaf087

Tip_names_suppl_fig3_ycaf087

## Data Availability

The datasets with gene content, phylogenetic trees, and code to replicate the predictive analyses are available on Github (https://github.com/RedondoMA/AOA_gene_predictions). The NCBI and JGI accession ID of the 457 AOA genomes that were used in this study are provided in Supplementary Table S1. The alignment to build the reference phylogeny tree is available as separated fasta file in supplementary information. The phylogenetic eigenvector- and ancestral state reconstruction-based predictions for the taxa of the reference phylogeny are provided in Supplementary Tables S5 and S7. The complete sequence dataset of the case study is available in the NCBI Short Read Archive under BioProject Accession no. PRJNA436119.
