# Supplementary figures and images for: Predicting gene distribution in ammonia-oxidizing archaea using phylogenetic signals
Source: ISME Commun. 2025 May 23;5(1):ycaf087. doi: 10.1093/ismeco/ycaf087 (PMC12254950; doi:10.1093/ismeco/ycaf087)

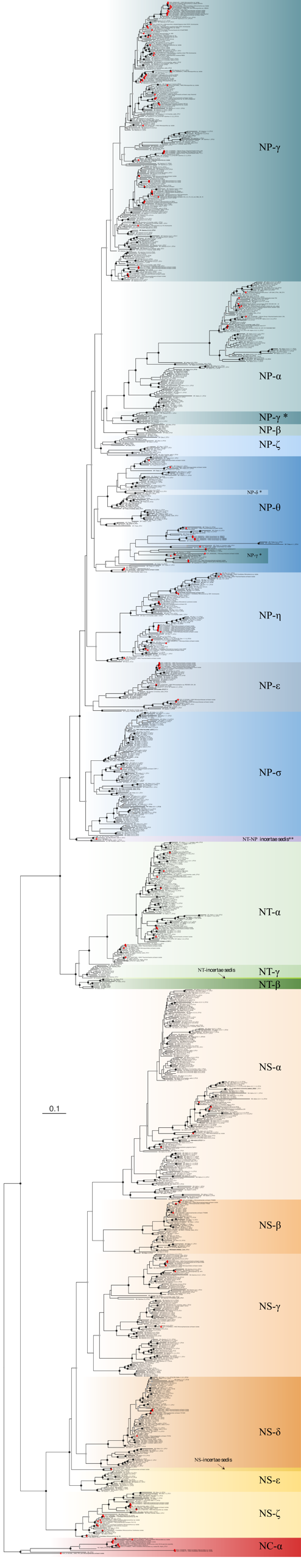

Supplement: Supplemental_fig3_ref_phylogenytree_ycaf087 [file supplemental_fig3_ref_phylogenytree_ycaf087.pdf]
